# Adalimumab serum levels and antidrug antibodies towards adalimumab in peripheral spondyloarthritis: no association with clinical response to treatment or with disease relapse upon treatment discontinuation

**DOI:** 10.1186/ar4675

**Published:** 2014-07-29

**Authors:** Jacqueline E Paramarta, Dominique L Baeten

**Affiliations:** Department of Clinical Immunology and Rheumatology, Academic Medical Center/University of Amsterdam, Meibergdreef 9 1105 AZ, Amsterdam, the Netherlands

## Abstract

**Introduction:**

In this study, we evaluated the clinical relevance of serum drug levels and antidrug antibodies (ADAbs) with regard to response to treatment, as well as to relapse upon treatment discontinuation, in peripheral spondyloarthritis (pSpA) patients treated with adalimumab.

**Methods:**

The study included 26 pSpA patients treated with adalimumab for either 12 weeks (*n* = 12) or 24 weeks (*n* = 14) in a randomized controlled trial. Patients achieving inactive disease measured by Ankylosing Spondylitis Disease Activity Score (ASDAS) at the end of the treatment period were classified as responders. Clinical characteristics, serum trough adalimumab levels and ADAbs were assessed at the end of the treatment period and at follow-up (upon relapse or, in absence of relapse, at 16 weeks after discontinuation).

**Results:**

Serum adalimumab levels measured 2 weeks after the last adalimumab administration ranged from <0.002 to 23.0 μg/ml, with a median of 11.5 μg/ml. These levels were associated with neither response to treatment or disease activity measurements at the end of treatment nor with the occurrence of relapse and time to relapse after discontinuation of treatment. Antiadalimumab ADAbs were present in 23% of the patients at end of treatment and in 35% at follow-up after treatment discontinuation, indicating that ADAbs were masked by the presence of the drug in some patients. However, ADAbs at the end of treatment and at follow-up were not different between responders and nonresponders and were not associated with relapse upon discontinuation of treatment.

**Conclusions:**

There is no clear association between adalimumab serum levels or antiadalimumab ADAbs with clinical response to treatment or with relapse upon treatment discontinuation in pSpA.

**Trial registration:**

Netherlands Trial Register ID: NTR1806 (registered 7 May 2009)

## Introduction

Tumour necrosis factor (TNF) inhibition is a highly effective treatment for axial and peripheral spondyloarthritis (SpA) [1–5]. However, a significant proportion of patients fails to respond or does not tolerate the treatment because of side effects. The reasons for nonresponse or for intolerance are multiple, potentially including the development of antidrug antibodies (ADAbs) directed towards the TNF blocker. It has been proposed that ADAbs may reduce therapeutic responses, either by increasing the clearance of the TNF inhibitor [6] or by direct neutralisation of the functional part of the drug [7]. Accordingly, authors of recent reviews have suggested that monitoring of serum drug levels and ADAbs would be a promising tool for personalised cost-effective usage of biological therapies in immune-mediated inflammatory diseases (IMIDs) [8, 9].

Most studies on the immunogenicity of TNF blockers have been performed in rheumatoid arthritis and Crohn’s disease. In SpA, the available studies on immunogenicity have yielded conflicting results. Regarding infliximab and adalimumab, some research groups have reported that ADAbs towards these TNF inhibitors are associated with decreased clinical response and increased risk of hypersensitivity reactions [10–16], whereas others have not found this association and have even concluded that serum anti-TNF drug levels are not associated with response to treatment [17, 18]. For etanercept, these ADAbs have not been detected, and it has been shown that the serum drug levels are similar in responders and nonresponders [19]. Recently, golimumab ADAbs did not appear to have a major role in treatment success or failure [20, 21]. Moreover, the authors of a recent meta-analysis of anti-TNF ADAbs in various IMIDs concluded that there was no relevant association of ADAbs with efficacy in SpA [22]. This corresponds to the clinical experience that treatment failure after TNF blockade is similar among the various TNF blockers [23, 24], both the ones which do and those that do not cause the development of ADAbs according to the results of the aforementioned studies [10–21].

These conflicting results may be related to the diversity of methods used and the timing of the measurement to evaluate the ADAbs, as well as to the fact that the presence of detectable serum drug levels may mask the detection of ADAbs [8, 9]. The latter issue can be avoided by the use of novel assay methods [25] and/or by assessing the ADAbs several weeks after stopping the TNF inhibitor. In this study, we assessed the potential clinical relevance of serum drug levels and ADAbs measured at the end of the treatment period as well as at a drug-free follow-up examination after a double-blind, placebo-controlled, randomized clinical trial (RCT) with adalimumab in patients with peripheral SpA (pSpA) [5]. We correlated these serum levels with both clinical response at the end of treatment and relapse upon discontinuation of the TNF inhibitor.

## Methods

### Study patients

Twenty-six patients from our RCT with adalimumab in peripheral arthritis in SpA who did not fulfil the criteria for ankylosing spondylitis (AS) or psoriatic arthritis (PsA) [5] were included in this study. The patients fulfilled the European Spondyloarthritis Study Group (ESSG) criteria [26] and/or the Amor *et al*. criteria [27] and had active disease at the time of inclusion. They were treated with either placebo (*n* = 12) or adalimumab (*n* = 14) for 12 weeks, followed by a 12-week open-label phase with adalimumab for all patients [5]. After this period, adalimumab was discontinued. Patients were allowed to continue treatment with nonsteroidal anti-inflammatory drugs (NSAIDs), corticosteroids (≤10 mg/day prednisone or equivalent), methotrexate and sulfasalazine at a stable dosage throughout the study. After discontinuation of the TNF inhibitor, patients were prospectively followed for 16 weeks and seen for a relapse visit upon worsening of symptoms or, in the absence of relapse, at the 16-week follow-up visit. The following disease activity parameters were measured: patient’s and physician’s global assessment of disease activity, 68/66 tender joint count (TJC) and swollen joint count (SJC), Bath Ankylosing Spondylitis Disease Activity Index (BASDAI) score, Ankylosing Spondylitis Disease Activity Score (ASDAS), erythrocyte sedimentation rate (ESR) and C-reactive protein (CRP) level. Responders were defined as patients achieving inactive disease based on ASDAS at the end of treatment [28]. Relapse was defined as an increase of at least one swollen joint or an increase by at least two points in the patient’s or physician’s global assessment of disease activity or BASDAI score [29]. Fourteen patients (53.8%) reached ASDAS-defined inactive disease at the end of the treatment period. Nineteen patients (73.1%) had a disease relapse within 16 weeks after discontinuation of adalimumab, with a mean time to relapse of 10.0 ± 3.2 weeks. The characteristics of the patients have been published previously [29]. Written informed consent was obtained from each patient before study-related procedures were performed, and the study was approved by the Medical Ethics Committee of the Academic Medical Center/University of Amsterdam.

### Assessment of serum adalimumab levels

Serum samples were obtained 2 weeks after the last adalimumab administration (*n* = 26) and at follow-up (upon relapse or 16 weeks after discontinuation in the absence of relapse (*n* = 25). Trough serum adalimumab concentrations were measured using an enzyme-linked immunosorbent assay (ELISA) developed by Wolbink *et al*. as previously described and as accredited by the Dutch Accreditation Council/Dutch Accreditation Board for Medical Laboratories (RvA/CCKL) according to the International Standardization Organization (ISO) guideline ISO17025 [30]. The detection limit of the assay is approximately 0.001 μg/ml; serum adalimumab levels <5.0 μg/ml were designated low, as previously described [15, 30].

### Assessment of antibodies against adalimumab

The same serum samples were analysed by radioimmunoassay (Sanquin) to detect the presence of antiadalimumab antibodies as previously described [30]. Dilution of 1 μl of serum in phosphate-buffered saline/0.3% bovine serum albumin (*pro analysi* buffer) was followed by overnight incubation with 1 mg of Sepharose-immobilized protein A (GE Healthcare, Chalfont St Giles, UK) in a final volume of 800 μl. Next, the samples were washed with phosphate-buffered saline 0.005% polysorbate. The antiadalimumab binding was determined by overnight incubation with 20,000 disintegrations per minute (dpm (approximately 1 ng)) of ^125^I-labeled F(ab)_2_ adalimumab fragments diluted in Freeze buffer (Sanquin, Amsterdam, the Netherlands). Unbound label was removed by washing, and protein A–bound radioactivity was measured. Antiadalimumab levels were expressed in arbitrary units (AU; 1 AU ≈ 12 ng) using a serum containing antiadalimumab as standard. The mean cutoff value derived from 100 healthy donors was set at 12 AU/ml.

### Statistical analysis

The data are presented as medians and interquartile ranges (IQRs). Mann–Whitney *U* tests were used to compare differences in serum levels in cases of unpaired samples, and Wilcoxon signed-rank tests were performed in cases of paired samples. χ^2^ tests were used for categorical variables. Logistic regression analyses were conducted to examine associations between the ASDAS response, relapse status and trough serum adalimumab and antiadalimumab ADAb levels. Correlations between the serum measurements and the clinical disease activity measurements were assessed using Spearman’s correlation tests. All statistical tests were two-sided, and *P*-values <0.05 were considered statistically significant.

## Results

### Clinical response to treatment and relapse after anti-TNF treatment discontinuation are independent of trough serum adalimumab levels

**Figure 1:**
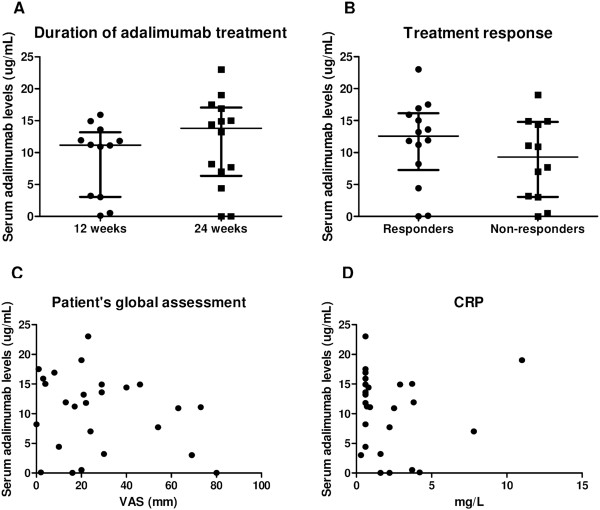
**Clinical response to treatment is independent of trough serum adalimumab levels.** Trough serum adalimumab levels at the end of treatment (2 weeks after the last injection) were not different between patients treated with 12 or 24 weeks of adalimumab **(A)** or between responders and nonresponders **(B)** Median (interquartile range). Also, there was no correlation between these drug levels and clinical disease activity parameters such as patient’s global assessment of disease activity measured on a 100-mm visual analogue scale (VAS) **(C)** or C-reactive protein (CRP) level **(D)**.

**Figure 2:**
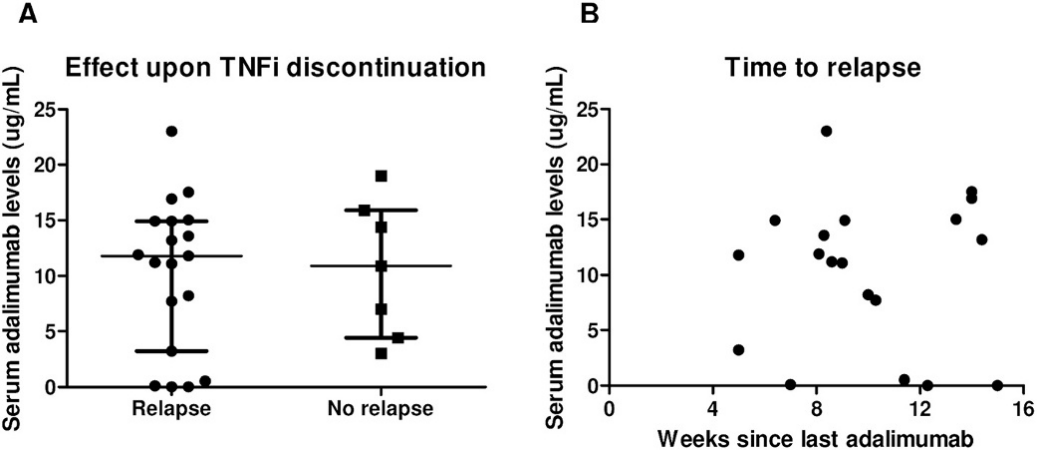
**Relapse after treatment discontinuation is independent of trough serum adalimumab levels.** Trough serum adalimumab levels at the end of treatment (2 weeks after the last injection) were similar between patients who did and those who did not relapse after discontinuation of the tumour necrosis factor inhibitor (TNFi) **(A)** Median (interquartile range). Neither was there a correlation between the drug levels and time to relapse **(B)**.

Trough serum adalimumab levels at the end of the treatment period (2 weeks after the last injection) ranged from <0.002 to 23.0 μg/ml (median = 11.5 μg/ml). Seven patients (26.9%) had serum adalimumab levels <5.0 μg/ml. Levels were not different between patients treated with adalimumab for 12 or 24 weeks (*P* = 0.292) (Figure 1A). There were no significant differences in trough serum adalimumab levels between responders (median = 12.6 (IQR = 7.3 to 16.2) μg/ml) and nonresponders (9.3 (3.1 to 14.5) μg/ml) as defined by the achievement of inactive disease defined by ASDAS (*P* = 0.237) (Figure 1B). Serum adalimumab levels also did not correlate with end-of-study disease activity parameters such as patient’s global assessment (Figure 1C) and physician’s global assessment of disease activity, TJC, SJC, BASDAI score, ASDAS, ESR (data not shown) and CRP level (Figure 1D). Moreover, adalimumab levels at the end of treatment were not different between patients with vs. without subsequent relapse upon discontinuation of therapy (*P* = 0.931) (Figure 2A) and were not correlated with time to relapse (*P* = 0.984) (Figure 2B). Taken together, these data indicate that the amplitude and/or duration of clinical response to adalimumab in pSpA patients are not related to trough serum adalimumab levels.

### Clinical response to treatment and relapse after anti-TNF treatment discontinuation are independent of presence of antiadalimumab antidrug antibodies

**Figure 3:**
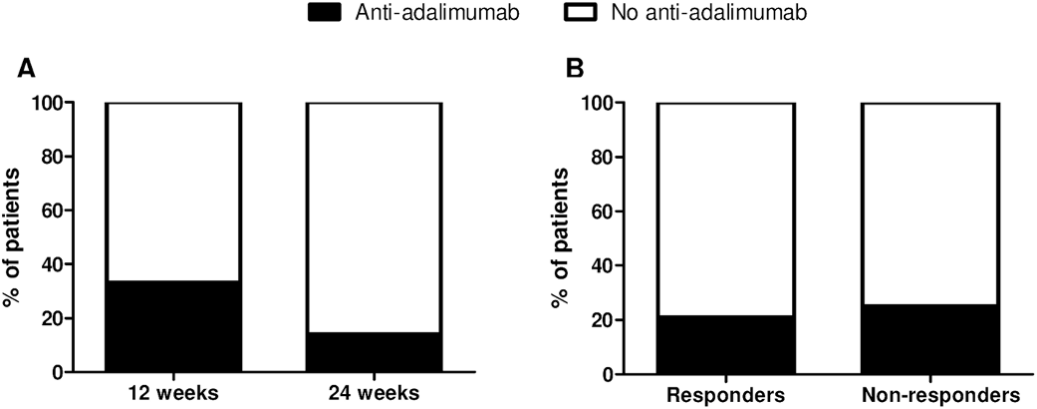
**Clinical response to treatment is independent of the presence of antiadalimumab antidrug antibodies.** Antiadalimumab antidrug antibodies (ADAbs) at the end of treatment, 2 weeks after the last injection, were not different between patients treated with 12 vs. 24 weeks of adalimumab **(A)** or between responders vs. nonresponders **(B)**.

**Figure 4:**
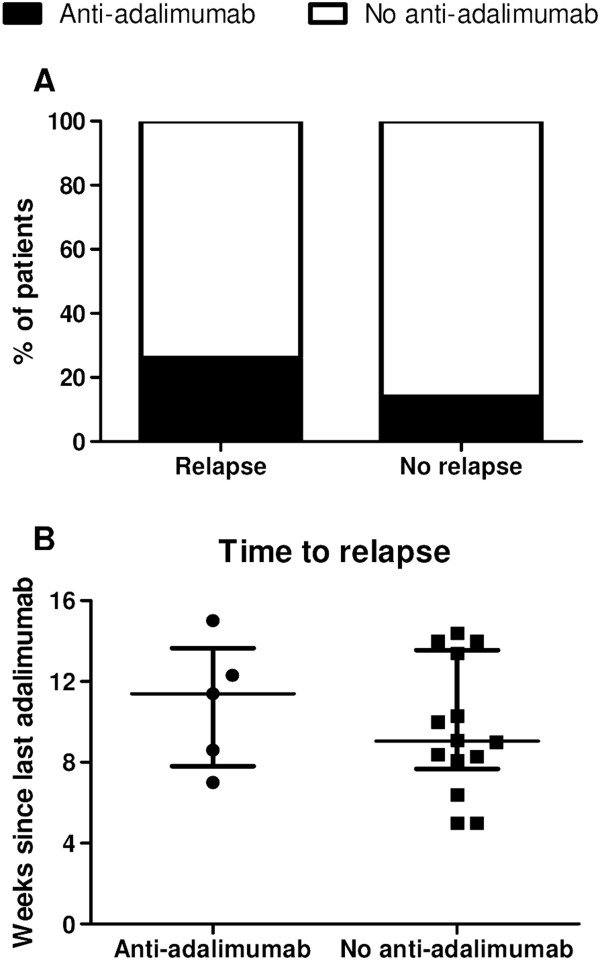
**Relapse after treatment discontinuation is independent of the presence of antiadalimumab antidrug antibodies.** Antiadalimumab antidrug antibodies (ADAbs) at the end of treatment, 2 weeks after the last injection, were not different between patients who did vs. those who did not relapse after discontinuation of adalimumab **(A)**. There was no difference in time to relapse in patients who tested positive vs. those who tested negative for antiadalimumab ADAbs **(B)** Median (interquartile range).

At the end of the treatment period, 6 (23.1%) of 26 patients tested positive for serum antiadalimumab ADAbs: 4 were clearly positive, with titres ranging from 89 to 2,320 AU/ml, and 2 were borderline positive, both with a titre of 15 AU/ml. The presence of detectable antiadalimumab ADAb levels was similar between patients treated with adalimumab for 12 weeks (4 (33.3%) of 12 patients) or for 24 weeks (2 (14.3%) of 14 patients) (*P* = 0.250) (Figure 3A) and between responders (3 (21.4%) of 14 patients) and nonresponders (3 (25.0%) 12 patients) (*P* = 0.829) (Figure 3B). The antiadalimumab ADAb titres did not correlate with the various disease activity measurements (data not shown). Finally, the number of patients who tested positive for antiadalimumab ADAbs was not different between those with vs. without subsequent relapse upon discontinuation of therapy (*P* = 0.518) (Figure 4A), nor was there a difference in time to relapse (*P* = 0.488) (Figure 4B). As for the trough serum adalimumab levels, our data do not provide any evidence that antiadalimumab ADAbs have a significant impact on the amplitude and/or the duration of clinical response to adalimumab in pSpA.

### Antiadalimumab ADAbs can be masked by presence of adalimumab; ‘unmasked’ antiadalimumab ADAbs do not correlate with clinical response to treatment

**Figure 5:**
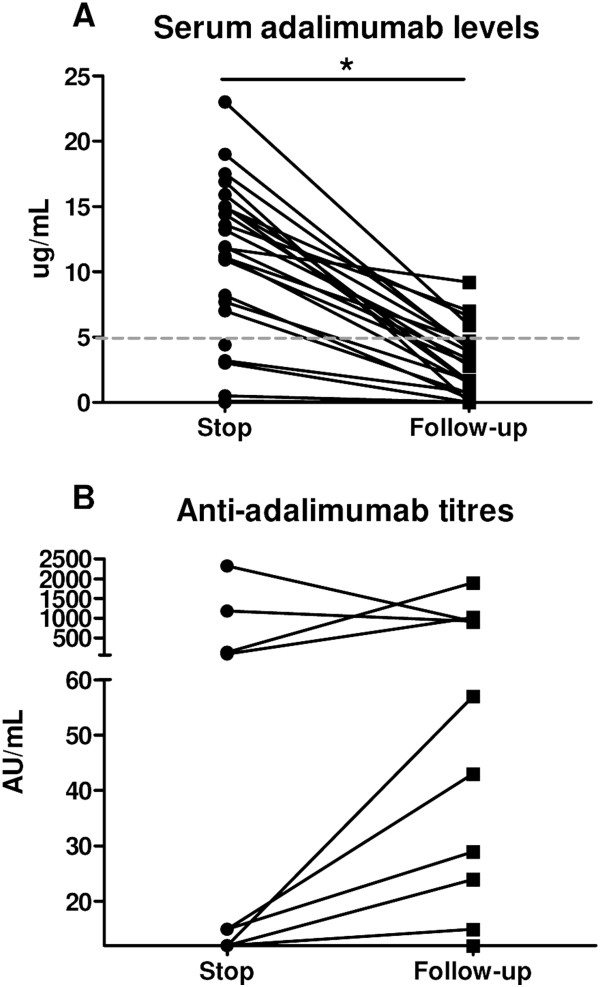
**Antiadalimumab antidrug antibodies can be masked by the presence of adalimumab in serum.** Trough serum adalimumab levels **(A)** and antiadalimumab antidrug antibody (ADAb) titres **(B)** at the end of treatment (2 weeks after the last injection) and at follow-up (upon relapse or, in absence of relapse, at 16 weeks after discontinuation). *P < 0.05 assessed by Wilcoxon signed-rank test.

As several factors can bias the measurement and/or interpretation of ADAbs, we conducted additional analyses to assess the potential clinical relevance of these antibodies. First, there was a negative correlation between the antiadalimumab ADAb titres and serum trough adalimumab levels (*R* = -0.709, *P* < 0.001). This may be explained by the fact that ADAbs contribute to the clearance of the drug or, alternatively, by the fact that serum adalimumab levels may interfere with the measurement of the ADAbs [8, 9]. To investigate the latter possibility, we obtained additional serum samples from 25 of 26 patients at a median follow-up of 10.1 (IQR = 8.3 to 14.0) weeks after interruption of the adalimumab treatment. At this time point, the median serum adalimumab level was 1.7 (IQR = 0.2 to 4.4) μg/ml, which was significantly lower than the level at the end of treatment (*P* < 0.0001) (Figure 5A). Measurement of antiadalimumab ADAbs at the same time point, which allowed us to exclude interference of circulating adalimumab levels, showed that the four patients with high ADAbs at the end of treatment maintained high ADAb levels after interruption of treatment, whereas the titres tended to increase over time in the three patients with low ADAb titres at the end of treatment (Figure 5B). Additionally, three patients without detectable ADAbs at the end of treatment had titres between 15 and 57 AU/ml at follow-up, confirming that low to intermediate levels of ADAbs may be partially masked by circulating adalimumab. However, these ‘unmasked’ antiadalimumab ADAbs were not different between responders (33.3% positive for ‘unmasked’ antiadalimumab) and nonresponders (40.0% positive for ‘unmasked’ antiadalimumab) (*P* = 0.734) or between patients who did vs. those who did not experience relapse after treatment discontinuation (42.1% and 16.7%, respectively; *P* = 0.258). The titres of the ‘unmasked’ ADAbs were not correlated with either the various disease activity measurements or the time to relapse (data not shown).

**Figure 6:**
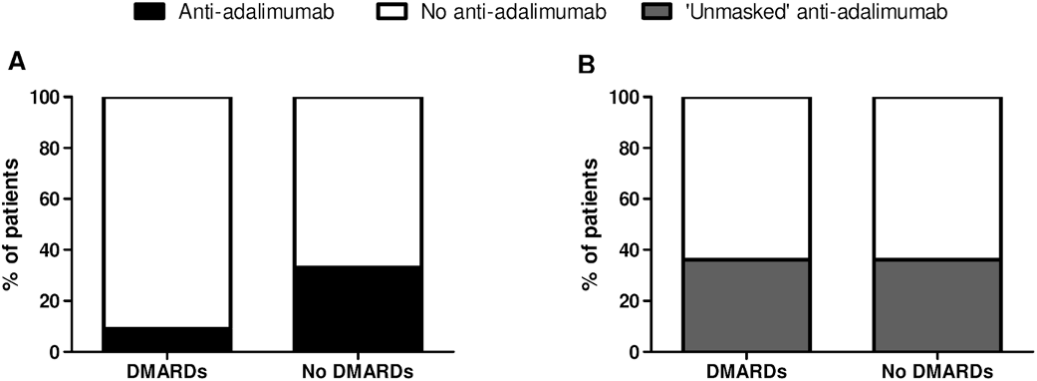
**The effect of disease-modifying antirheumatic drugs on antiadalimumab antidrug antibodies.** At the end of treatment (2 weeks after the last injection), antiadalimumab antidrug antibodies (ADAbs) were less often observed in patients who used concomitant disease-modifying antirheumatic drugs (DMARDs) compared than in patients who did not use any DMARDs, although this finding was not statistically significant **(A)**. However, this difference disappeared when we assessed the ‘unmasked’ antiadalimumab ADAbs at follow-up **(B)**.

Second, we investigated the effect of concomitant treatment with disease-modifying antirheumatic drugs (DMARDs) on ADAbs because this has been described to reduce the frequency of ADAb formation [8, 9, 14, 31]. Although numerically different, there was no statistical difference in antiadalimumab ADAb positivity between patients taking DMARDs (5 (33.3%) of 15 patients,) vs. patients not taking DMARDs (1, (9.1%) of 11 patients) (*P* = 0.147) (Figure 6A). Strikingly, this difference was no longer present when we analysed the ‘unmasked’ antiadalimumab ADAbs: 5 (35.7%) of 14 patients who were positive in the no-DMARDs group vs. 4 (36.4%) of 11 patients in the DMARDs group (*P* = 0.973) (Figure 6B).

### Adalimumab levels and antiadalimumab antidrug antibodies in patients with generalized drug reactions

Two patients developed a generalized skin reaction upon initiation of adalimumab during the study. Both patients were able to continue adalimumab treatment after administration of topical corticosteroids and treatment with oral antihistamine. One of these patients had a low serum trough adalimumab level (<0.002 μg/ml) and tested positive for antiadalimumab ADAbs with a titre of 2,320 AU/ml. The second patient had a serum trough adalimumab level of 17.5 μg/ml and tested negative for antiadalimumab ADAbs both at the end of the study and at follow-up after interruption of treatment. Neither of these two patients used concomitant DMARDs.

## Discussion

In this study, we assessed the potential relevance of serum drug levels and ADAbs on various aspects of clinical response to adalimumab treatment in pSpA. The major findings are that (1) trough serum adalimumab levels were heterogeneous but did not correlate with clinical response to treatment or relapse after anti-TNF treatment discontinuation, (2) antiadalimumab ADAbs were found in one-fourth of the patients but also did not correlate with clinical response to treatment or relapse after discontinuation of the TNF inhibitor and (3) low-titre ADAbs could be masked by circulating adalimumab, but ‘unmasked’ ADAbs showed no clear relationship with clinical efficacy.

More and more research is being done to address the immunogenicity of TNF inhibitors in various IMIDs, including SpA, because the development of ADAbs towards these TNF inhibitors are assumed to play a major role in loss of response to treatment. The mechanism behind this is thought to be either an increased clearance of the drug or neutralization of the active component of the compound [6, 7]. This hypothesis is supported by researchers in several studies of infliximab and adalimumab who reported that treatment failure occurred more often in patients who tested positive for ADAbs [10–14]. However, there is also evidence which is not in line with this concept; in other studies of infliximab, investigators did not find a relation between ADAbs and response to treatment [17, 18]. Moreover, in one of the studies in which researchers found serum trough infliximab levels to be significantly higher in responders than in nonresponders, although statistical significance was reached, the difference between these groups was very low (8.2 μg/ml vs. 6.3 μg/ml, respectively) [12]. Whether such a small difference is really clinically relevant is questionable. ADAbs towards etanercept have not been found, nor is there an association between serum drug levels and clinical effect [19]. Likewise, for golimumab, there is no clear relation between ADAbs and clinical efficacy [20, 21]. Furthermore, in a recent meta-analysis of various TNF inhibitors [22] and the clinical experience of there being no difference in efficacy or drug survival between the various TNF inhibitors [23, 24], the authors also questioned the clinical relevance of anti-TNF ADAbs.

Several factors could be examined to develop an explanation of these differences between the various studies. First, not all TNF inhibitors are assumed to be equally immunogenic. For example, the soluble dimeric fusion protein etanercept has a less immunogenic structure because only the fusion part of the molecule can contain immunogenic epitopes. Also, it is administered more frequently than the other TNF inhibitors, thereby possibly creating more drug interference in ADAb detection [19]. However, this does not explain why researchers in different studies of the same TNF inhibitor (for example, infliximab) have come to different conclusions [10–14, 17, 18]. Second, there may be differences among the different SpA subtypes. However, even in studies of both the same disease and the same TNF inhibitor (for example, infliximab in AS), contradicting conclusions have been drawn [10–12, 17, 18]. Hence, this could also not explain why we and others [21] did not find a clinical association with anti-TNF drug levels or ADAbs in pSpA, whereas researchers in another study did conclude that these factors had clinical relevance [15]. Third, there is some variation in the size and duration of the studies, but this did not influence the results with regard to whether ADAbs did or did not have clinical relevance [10–21]. Fourth, the use of DMARDs, especially methotrexate, has been described to decrease the immunogenicity of TNF inhibitor trough, a mechanism which is not yet understood [8, 9, 14, 31]. However, this is not in line with the finding that the addition of methotrexate in the management of SpA does not have an impact on the efficacy of the treatment [23, 24, 32] or on drug survival of the TNF inhibitor [23, 24]. In our present study, we indeed found fewer of ADAbs in patients using DMARDs; however, this difference was no longer present when we analysed the ‘unmasked ADAbs’. Fifth, the detection of ADAbs is also influenced by the assay used. However, the method used in our present study is the same as that used in other studies [15, 16], making it unlikely that this is the explanation for the differences in results. Seventh, the timing of the samples also influences the measurement of ADAbs because the assays are sensitive to drug interference, even when measured before the next administration of the drug, when the drug levels are the lowest [8, 9]. Indeed, we show here that when antiadalimumab ADAbs were measured at follow-up, after discontinuation of the TNF inhibitor, more patients tested positive than when antiadalimumab ADAbs were measured at the end of treatment. Previous researchers have reported that anti-TNF ADAb titres can decrease and increase over time, and vice versa [10, 15, 33], causing a gradual increase in incidence over time when ADAb status is presented cumulatively, but not when assessed at each time point independently [33]. This shows that the timing of the measurement can influence the interpretation of ADAb status, making it very difficult to make strong conclusions about the relationship with clinical response and to apply ADAb measurement in clinical practise.

The limited number of patients is a limitation of our study. We thus cannot exclude that clinical correlation with serum adalimumab levels and/or ADAbs would be found in larger patient cohorts. However, this would imply that this association is weak and thus not relevant anyway for treatment monitoring in individual patients. Also, in one small study in which researchers investigated whether the serum trough infliximab levels modified therapeutic decisions in the management of AS, no improvement in the control of disease activity was found [34]. Similar efficacy and drug survival of TNF inhibitors—those that induce ADAbs and those which do not [23, 24] —also raise questions about the relevance of testing the immunogenicity of these drugs. Although testing immunogenicity in clinical trials is standard practise and may yield interesting scientific insights, the real added value of the presence or absence of ADAbs in an individual patient in clinical practise remains to be demonstrated.

## Conclusions

The link between either serum adalimumab levels or antiadalimumab ADAbs and clinical response or relapse is not as strong as previously assumed. This argues against the use of these parameters in monitoring drug efficacy. Although treatment with adalimumab and other TNF inhibitors is very successful overall in SpA, future research has to be done to unravel factors which can explain differences in clinical response to further improve disease management by developing a personalized and more cost-effective approach.

## Abbreviations

ADAbs: Antidrug antibodies
AS: Ankylosing spondylitis
ASDAS: Ankylosing Spondylitis Disease Activity Score
AU: Arbitrary units
BASDAI: Bath Ankylosing Spondylitis Disease Activity Index
CRP: C-reactive protein
DMARD: Disease-modifying antirheumatic drug
ELISA: Enzyme-linked immunosorbent assay
ESR: Erythrocyte sedimentation rate
ESSG: European Spondyloarthritis Study Group
IMID: Immune-mediated inflammatory disease
IQR: Interquartile range
NSAID: Nonsteroidal anti-inflammatory drug
PsA: Psoriatic arthritis
pSpA: Peripheral spondyloarthritis
RCT: Randomized controlled trial
SJC: Swollen joint count
SpA: Spondyloarthritis
TJC: Tender joint count
TNF: Tumour necrosis factor.

## References

[1] Van den Bosch F, Kruithof E, Baeten D, Herssens A, De Keyser F, Mielants H, Veys EM (2002). Randomized double-blind comparison of chimeric monoclonal antibody to tumor necrosis factor α (infliximab) versus placebo in active spondylarthropathy. Arthritis Rheum.

[2] Braun J, Brandt J, Listing J, Zink A, Alten R, Golder W, Gromnica-Ihle E, Kellner H, Krause A, Schneider M, Sörensen H, Zeidler H, Thriene W, Sieper J (2002). Treatment of active ankylosing spondylitis with infliximab: a randomised controlled multicentre trial. Lancet.

[3] Antoni C, Krueger GG, de Vlam K, Birbara C, Beutler A, Guzzo C, Zhou B, Dooley LT, Kavanaugh A, for the IMPACT 2 investigators (2005). Infliximab improves signs and symptoms of psoriatic arthritis: results of the IMPACT 2 trial. Ann Rheum Dis.

[4] Sieper J, van der Heijde D, Dougados M, Mease PJ, Maksymowych WP, Brown MA, Arora V, Pangan AL (2013). Efficacy and safety of adalimumab in patients with non-radiographic axial spondyloarthritis: results of a randomised placebo-controlled trial (ABILITY-1). Ann Rheum Dis.

[5] Paramarta JE, De Rycke L, Heijda TF, Ambarus CA, Vos K, Dinant HJ, Tak PP, Baeten DL (2013). Efficacy and safety of adalimumab for the treatment of peripheral arthritis in spondyloarthritis patients without ankylosing spondylitis or psoriatic arthritis. Ann Rheum Dis.

[6] Van der Laken CJ, Voskuyl AE, Roos JC, Stigter Van Walsum M, de Groot ER, Wolbink G, Dijkmans BA, Aarden LA (2007). Imaging and serum analysis of immune complex formation of radiolabelled infliximab and anti-infliximab in responders and non-responders to therapy for rheumatoid arthritis. Ann Rheum Dis.

[7] van Schouwenburg PA, van de Stadt LA, de Jong RN, van Buren EE, Kruithof S, de Groot E, Hart M, van Ham SM, Rispens T, Aarden L, Wolbink GJ, Wouters D (2013). Adalimumab elicits a restricted anti-idiotypic antibody response in autoimmune patients resulting in functional neutralisation. Ann Rheum Dis.

[8] Vincent FB, Morand EF, Murphy K, Mackay F, Mariette X, Marcelli C (2013). Antidrug antibodies (ADAb) to tumour necrosis factor (TNF)-specific neutralising agents in chronic inflammatory diseases: a real issue, a clinical perspective. Ann Rheum Dis.

[9] Garcês S, Demengeot J, Benito-Garcia E (2013). The immunogenicity of anti-TNF therapy in immune-mediated inflammatory diseases: a systematic review of the literature with a meta-analysis. Ann Rheum Dis.

[10] Arends S, Lebbink HR, Spoorenberg A, Bungener LB, Roozendaal C, van der Veer E, Houtman PM, Griep EN, Limburg PC, Kallenberg CG, Wolbink GJ, Brouwer E (2010). The formation of autoantibodies and antibodies to TNF-α blocking agents in relation to clinical response in patients with ankylosing spondylitis. Clin Exp Rheumatol.

[11] de Vries MK, Wolbink GJ, Stapel SO, de Groot ER, Dijkmans BA, Aarden LA, van der Horst-Bruinsma IE (2007). Inefficacy of infliximab in ankylosing spondylitis is correlated with antibody formation. Ann Rheum Dis.

[12] de Vries MK, Wolbink GJ, Stapel SO, de Vrieze H, van Denderen JC, Dijkmans BA, Aarden LA, van der Horst-Bruinsma IE (2007). Decreased clinical response to infliximab in ankylosing spondylitis is correlated with anti-infliximab formation. Ann Rheum Dis.

[13] Ducourau E, Mulleman D, Paintaud G, Miow Lin DC, Lauféron F, Ternant D, Watier H, Goupille P (2011). Antibodies toward infliximab are associated with low infliximab concentration at treatment initiation and poor infliximab maintenance in rheumatic diseases. Arthritis Res Ther.

[14] Plasencia C, Pascual-Salcedo D, Nuño L, Bonilla G, Villalba A, Peiteado D, Díez J, Nagore D, del Agua AR, Moral R, Martin-Mola E, Balsa A (2012). Influence of immunogenicity on the efficacy of longterm treatment of spondyloarthritis with infliximab. Ann Rheum Dis.

[15] van Kuijk AW, de Groot M, Stapel SO, Dijkmans BA, Wolbink GJ, Tak PP (2010). Relationship between the clinical response to adalimumab treatment and serum levels of adalimumab and anti-adalimumab antibodies in patients with psoriatic arthritis. Ann Rheum Dis.

[16] de Vries MK, Brouwer E, van der Horst-Bruinsma IE, Spoorenberg A, van Denderen JC, Jamnitski A, Nurmohamed MT, Dijkmans BA, Aarden LA, Wolbink GJ (2009). Decreased clinical response to adalimumab in ankylosing spondylitis is associated with antibody formation. Ann Rheum Dis.

[17] Baraliakos X, Listing J, Rudwaleit M, Brandt J, Alten R, Burmester G, Gromnica-Ihle E, Haibel H, Schewe S, Schneider M, Sörensen H, Zeidler H, Visvanathan S, Sieper J, Braun J (2007). Safety and efficacy of readministration of infliximab after longterm continuous therapy and withdrawal in patients with ankylosing spondylitis. J Rheumatol.

[18] Krzysiek R, Breban M, Ravaud P, Prejean MV, Wijdenes J, Roy C, Henry YD, Barbey C, Trappe G, Dougados M, Emilie D, French Ankylosing Spondylitis Infliximab Network (2009). Circulating concentration of infliximab and response to treatment in ankylosing spondylitis: results from a randomized control study. Arthritis Rheum.

[19] de Vries MK, van der Horst-Bruinsma IE, Nurmohamed MT, Aarden LA, Stapel SO, Peters MJ, van Denderen JC, Dijkmans BA, Wolbink GJ (2009). Immunogenicity does not influence treatment with etanercept in patients with ankylosing spondylitis. Ann Rheum Dis.

[20] Inman RD, Davis JC, Heijde D, Diekman L, Sieper J, Kim SI, Mack M, Han J, Visvanathan S, Xu Z, Hsu B, Beutler A, Braun J (2008). Efficacy and safety of golimumab in patients with ankylosing spondylitis: results of a randomized, double-blind, placebo-controlled, phase III trial. Arthritis Rheum.

[21] Kavanaugh A, McInnes I, Mease P, Krueger GG, Gladman D, Gomez-Reino J, Papp K, Zrubek J, Mudivarthy S, Mack M, Visvanathan S, Beutler A (2009). Golimumab, a new human tumor necrosis factor α antibody, administered every four weeks as a subcutaneous injection in psoriatic arthritis: twenty-four-week efficacy and safety results of a randomized, placebo-controlled study. Arthritis Rheum.

[22] Maneiro JR, Salgado E, Gomez-Reino JJ (2013). Immunogenicity of monoclonal antibodies against tumor necrosis factor used in chronic immune-mediated inflammatory conditions: systematic review and meta-analysis. JAMA Intern Med.

[23] Glintborg B, Ostergaard M, Krogh NS, Andersen MD, Tarp U, Loft AG, Lindegaard HM, Holland-Fischer M, Nordin H, Jensen DV, Olsen CH, Hetland ML (2013). Clinical response, drug survival, and predictors thereof among 548 patients with psoriatic arthritis who switched tumor necrosis factor α inhibitor therapy: results from the Danish Nationwide DANBIO Registry. Arthritis Rheum.

[24] Glintborg B, Ostergaard M, Krogh NS, Dreyer L, Kristensen HL, Hetland ML (2010). Predictors of treatment response and drug continuation in 842 patients with ankylosing spondylitis treated with anti-tumour necrosis factor: results from 8 years’ surveillance in the Danish nationwide DANBIO registry. Ann Rheum Dis.

[25] van Schouwenburg PA, Bartelds GM, Hart MH, Aarden L, Wolbink GJ, Wouters D (2010). A novel method for the detection of antibodies to adalimumab in the presence of drug reveals "hidden" immunogenicity in rheumatoid arthritis patients. J Immunol Methods.

[26] Dougados M, van der Linden S, Juhlin R, Huitfeldt B, Amor B, Calin A, Cats A, Dijkmans B, Olivieri I, Pasero G, Veys E, Zeidler H (1991). The European Spondylarthropathy Study Group preliminary criteria for the classification of spondylarthropathy. Arthritis Rheum.

[27] Amor B, Dougados M, Mijiyawa M (1990). [Criteria of the classification of spondylarthropathies] [in French]. Rev Rhum Mal Osteoartic.

[28] Machado P, Landewé R, Lie E, Kvien TK, Braun J, Baker D, van der Heijde D, for the Assessment of SpondyloArthritis International Society (2011). Ankylosing Spondylitis Disease Activity Score (ASDAS): defining cut-off values for disease activity states and improvement scores. Ann Rheum Dis.

[29] Paramarta JE, Heijda TF, Baeten DL (2013). Fast relapse upon discontinuation of tumour necrosis factor blocking therapy in patients with peripheral spondyloarthritis. Ann Rheum Dis.

[30] Bartelds GM, Krieckaert CL, Nurmohamed MT, van Schouwenburg PA, Lems WF, Twisk JW, Dijkmans BA, Aarden L, Wolbink GJ (2011). Development of antidrug antibodies against adalimumab and association with disease activity and treatment failure during long-term follow-up. JAMA.

[31] Jani M, Barton A, Warren RB, Griffiths CE, Chinoy H (2014). The role of DMARDs in reducing the immunogenicity of TNF inhibitors in chronic inflammatory diseases. Rheumatology (Oxford).

[32] Fagerli KM, Lie E, van der Heijde D, Heiberg MS, Lexberg AS, Rødevand E, Kalstad S, Mikkelsen K, Kvien TK (2014). The role of methotrexate co-medication in TNF-inhibitor treatment in patients with psoriatic arthritis: results from 440 patients included in the NOR-DMARD study. Ann Rheum Dis.

[33] Hanauer SB, Wagner CL, Bala M, Mayer L, Travers S, Diamond RH, Olson A, Bao W, Rutgeerts P (2004). Incidence and importance of antibody responses to infliximab after maintenance or episodic treatment in Crohn’s disease. Clin Gastroenterol Hepatol.

[34] Méric JC, Mulleman D, Ducourau E, Lauféron F, Miow Lin DC, Watier H, Goupille P, Paintaud G (2011). Therapeutic drug monitoring of infliximab in spondyloarthritis: an observational open-label study. Ther Drug Monit.

